# *Listeria* sanitizer tolerance at use-level concentrations shows limited association with genetic loci

**DOI:** 10.1128/aem.01060-25

**Published:** 2026-01-20

**Authors:** Anna Sophia Harrand, Jordan Skeens, Laura Carroll, Renato Orsi, Martin Wiedmann, Samantha Bolten

**Affiliations:** 1 Department of Food Science, Cornell University 5922, Ithaca, New York, USA; 2 Department of Clinical Microbiology, SciLifeLab, Umeå University 8075, Umeå, Sweden; 3 Laboratory for Molecular Infection Medicine Sweden (MIMS), Umeå University 8075, Umeå, Sweden; 4 Umeå Centre for Microbial Research (UCMR), Umeå University 8075, Umeå, Sweden; 5 Integrated Science Lab (IceLab), Umeå University 8075, Umeå, Sweden; Anses, Maisons-Alfort Laboratory for Food Safety, Maisons-Alfort, France

**Keywords:** whole genome sequencing, produce safety, *Listeria*, peroxyacetic acid, benzalkonium chloride, sodium hypochlorite

## Abstract

The ability of *Listeria* to show reduced susceptibility to sanitizers commonly used in fresh produce packing and processing environments continues to be mentioned as a concern. We assessed the survival of 501 produce-associated *Listeria* isolates (328 *Listeria monocytogenes* [LM] and 173 *Listeria* spp. [LS]) after 30 s of exposure to benzalkonium chloride (BC, 300 ppm) and peroxyacetic acid (PAA, 80 ppm). A subset of 108 isolates was also exposed to sodium hypochlorite (NaOCl, 500 ppm) for 30 s. Isolates showed a range of log reductions, including 2.76–5.73 log for BC, 0.15–6.16 log for PAA, and 1.34–7.02 log for NaOCl; the variance of log reductions was significantly lower for BC compared to PAA and NaOCl. Cluster analysis on log reduction data identified four clusters, including one cluster of five LM isolates that showed reduced susceptibility to all three sanitizers. Log reductions of LS were significantly lower than LM after exposure to PAA, indicating reduced PAA susceptibility among LS. Whole genome sequence (WGS)-based characterization of all isolates revealed that the presence of known BC resistance genes (i.e., *bcrABC, mdrL*, and *sugE1/2*) was not significantly associated with log reductions to BC, and the presence of stress survival islet SSI-2 was not significantly associated with log reductions to PAA and NaOCl. Genome-wide association studies did not reveal any association of pangenome genes with phenotypic sanitizer susceptibility but identified several SNPs in core genes as associated with sanitizer susceptibility.

**IMPORTANCE:**

Despite frequently stated concerns about LM and LS with reduced susceptibility to sanitizers (which could facilitate persistence and increase risk of product contamination), there are limited data available on *Listeria* susceptibility to sanitizers used in produce packing and processing environments at their recommended use-level concentrations. Importantly, our data showed that reduced sanitizer susceptibility of *Listeria* is not linked to the presence of any previously reported sanitizer resistance genes. However, we identified a group of five LM isolates that showed reduced susceptibility to all three sanitizers tested; these isolates represented lineages I, II, and III. Combined, these data suggest that there are no distinct “sanitizer-resistant” clonal *Listeria* groups and that WGS data may not be particularly valuable for predicting sanitizer susceptibility at use-level concentrations. Moreover, the high variability of log reductions observed across all three sanitizers highlights the importance of considering log reduction variability, in addition to average log reduction, when assessing different sanitizers.

## INTRODUCTION

Reducing the risk of *Listeria monocytogenes* contamination of ready-to-eat (RTE) foods, such as fresh produce, which represented the top food vehicle category associated with listeriosis outbreaks in the U.S. between 2008 and 2017 (1), remains a top priority among public health and food industry stakeholders. Although *L. monocytogenes* can be introduced onto RTE foods at various stages of the food supply chain, the majority of listeriosis outbreaks are linked to contamination originating from the environment in food packing and/or processing facilities (1–3). However, controlling *L. monocytogenes* in food packing/processing environments can be challenging, given the propensity of *L. monocytogenes* to establish persistence in these types of environments for several months to years (4–7), which can, in turn, increase the risk of product contamination, recalls, and outbreaks.

Sanitization is an integral part of food safety management practices, and sanitizers are applied regularly in produce packing/processing environments to reduce microbial load on equipment and facility structures and mitigate the persistence of foodborne pathogens, including *Listeria* (8–10). While sanitizers are typically applied to food contact and non-food contact surfaces at manufacturer recommended use-level concentrations, there are certain instances in which sanitizers may be present in packing/processing environments below use-level concentrations. For example, use-level sanitizer concentrations can be diluted upon application in cases where (i) high organic loads from equipment and facility structures are not effectively removed prior to sanitizer application (i.e., during cleaning) (11), and (ii) improper sanitary equipment/facility design can make it difficult for sanitizers to access hard-to-reach sites (5). Thus, there is concern that *Listeria* present in produce and other food packing/processing environments can be repeatedly exposed to low sanitizer concentrations (e.g., <10 ppm for benzalkonium chloride (12–14)), which may allow for the selection of mutations and/or acquisition of resistance genes that confer enhanced sanitizer tolerance (15). For example, the presence of certain resistance genes that encode for efflux pumps and are located on the chromosome (e.g., *mdrL* (16)) or on mobile elements such as plasmids or transposons (e.g., *qacH* or *bcrABC* (13, 17–20)), as well as single-nucleotide polymorphisms (SNPs) in efflux pump repressor genes (e.g., *fepR* and *sugR* (21–23)), have been shown to confer increased tolerance of *Listeria* to low levels of quaternary ammonium compound (quat)-based sanitizers. However, there is limited understanding of the extent to which these resistance genes or mutations may confer enhanced survival of *Listeria* (referred to here as “reduced susceptibility”) following exposure to sanitizers at their manufacturer-recommended use-level concentrations.

In addition to genetic loci/SNPs with known association to sanitizer tolerance, identification of novel genetic markers that are associated with sanitizer tolerance phenotypes would be highly valuable. To this end, advanced analytical methods, such as genome-wide association studies (GWAS), can be used to identify genetic markers (e.g., genes and SNPs), within a defined population, that are statistically associated with a phenotype of interest (24). Although a few GWAS have been performed in previous studies to elucidate the genetic basis of *L. monocytogenes* phenotypes relevant to food safety, including acid adaptation (25) and growth at refrigeration temperatures (26), none, to the author’s knowledge, have been used to date to identify genetic markers associated with *Listeria* survival following exposure to use-level sanitizer concentrations.

To fill in the knowledge gaps detailed above, this study sought to characterize a large collection of produce-associated *L. monocytogenes* and other “non-*L*. *monocytogenes*” *Listeria* spp. (referred to henceforth as *L*. spp.) isolates both (i) phenotypically, through exposing individual isolates to use-level concentrations of three sanitizers commonly used for food packing/processing equipment and facility sanitation (i.e., sodium hypochlorite [NaOCl], peroxyacetic acid [PAA], and benzalkonium chloride [BC]) (27), and (ii) genetically, through whole genome sequencing (WGS)-based characterization. The aim was to provide sanitizer susceptibility levels and frequencies in produce-associated *L. monocytogenes* and *L*. spp. and to identify associations of known resistance genes and novel genes/SNPs that may confer reduced susceptibility to use-level concentrations of sanitizers.

## RESULTS

### Produce-associated *Listeria* isolate collection

We assembled a final collection of 501 produce-associated *Listeria* isolates that were used for phenotypic sanitizer susceptibility determination and WGS-based characterization. The *Listeria* isolates in our collection were obtained from diverse pre- and post-harvest locations and sources associated with growing, packing, and processing of fresh produce. The final collection comprised 328 *L*. *monocytogenes* (56 preharvest and 272 postharvest) and 173 *L*. spp. (29 preharvest and 144 postharvest) (Table S1). *L. monocytogenes* isolates represented lineages I (*n* = 96), II (*n* = 210), and III (*n* = 22), and 74 different clonal complexes (CC) based on the 7-gene multi-locus sequence typing (MLST) scheme (28). The 10 most common *L. monocytogenes* CCs were CC9 (*n* = 40), CC5 (*n* = 29), CC6 (*n* = 21), CC1 (*n* = 20), CC4 (*n* = 14), CC155 (*n* = 12), CC388 (*n* = 12), CC7 (*n* = 12), CC37 (*n* = 11), and CC369 (*n* = 10); five of these CCs (i.e., CC9, CC5, CC6, CC1, CC155, and CC7) are among the top globally distributed CCs (29). All *L*. spp. represented *sensu stricto* species, including *Listeria seeligeri* (*n* = 70), *Listeria innocua* (*n* = 59), *Listeria welshimeri* (*n* = 33), *Listeria marthii* (*n* = 6), and *Listeria ivanovii* (*n* = 5).

Although all 501 isolates were characterized for susceptibility to BC and PAA, only a subset of these isolates (*n* = 108) was characterized for susceptibility to NaOCl; this subset included 76 *L*. *monocytogenes* and 32 *L*. spp. isolates. These 76 *L*. *monocytogenes* isolates were selected to include one isolate per CC; if multiple sequence types (ST) represented a CC, an isolate was randomly chosen from the most common ST within the CC. In addition, if an isolate within a CC carried SSI-2, an isolate representing each genotype (i.e., positive or negative for SSI-2) was included. The 32 *L*. spp. were selected to include at least 15% of isolates available for each species (i.e., 14 *L*. *seeligeri*, 11 *L*. *innocua*, *5 L. welshimeri*, 1 *L*. *marthii*, and 5 *L*. *ivanovii*). In order to select *L*. spp. isolates that represented the highest phylogenetic diversity, WGS-based SNP trees were used to identify a similarity threshold that yielded the number of branches equal to the number of desired isolates for a given species (e.g., 14 *L*. *seeligeri* branches); for each branch, an isolate was selected randomly using a random number generator available in R.

### Exposure to quats achieves the most consistent log reductions with the least variability within the population

**Fig 1:**
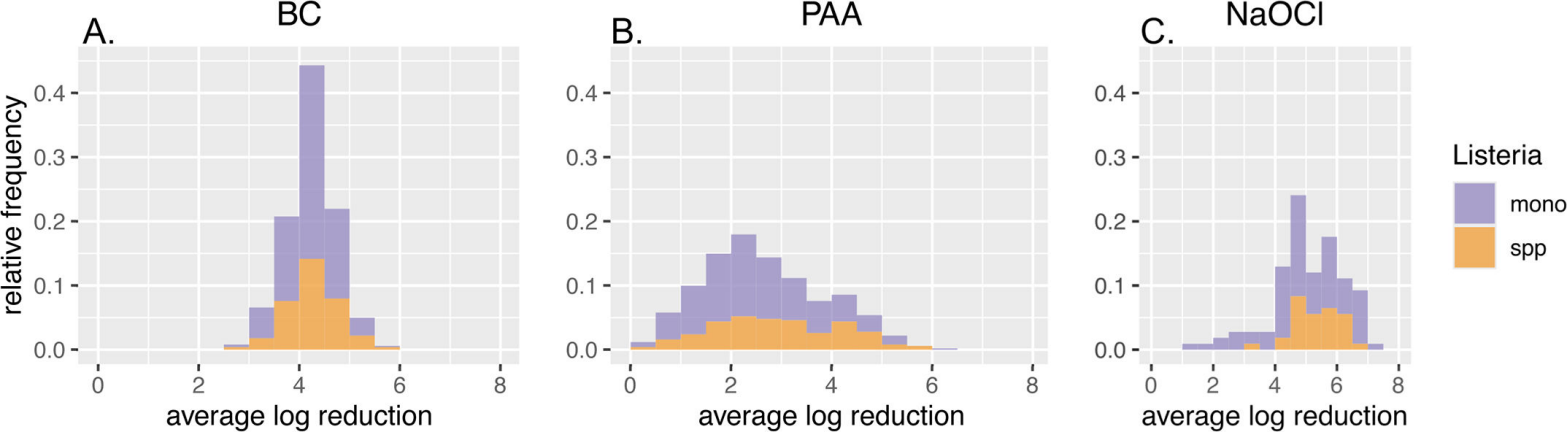
Histogram displaying average log reductions of *Listeria* isolates following exposure to sanitizers for 30 s. A total of 501 isolates were exposed to benzalkonium chloride (BC) at 300 ppm (**A**) and peroxyacetic acid (PAA) at 80 ppm (**B**); 108 isolates were exposed to sodium hypochlorite (NaOCl) at 500 ppm (**C**). Average log reductions of *L. monocytogenes* isolates (mono) are shown in purple, and average log reductions of *L*. spp. isolates (spp) are shown in orange.

**Fig 2:**
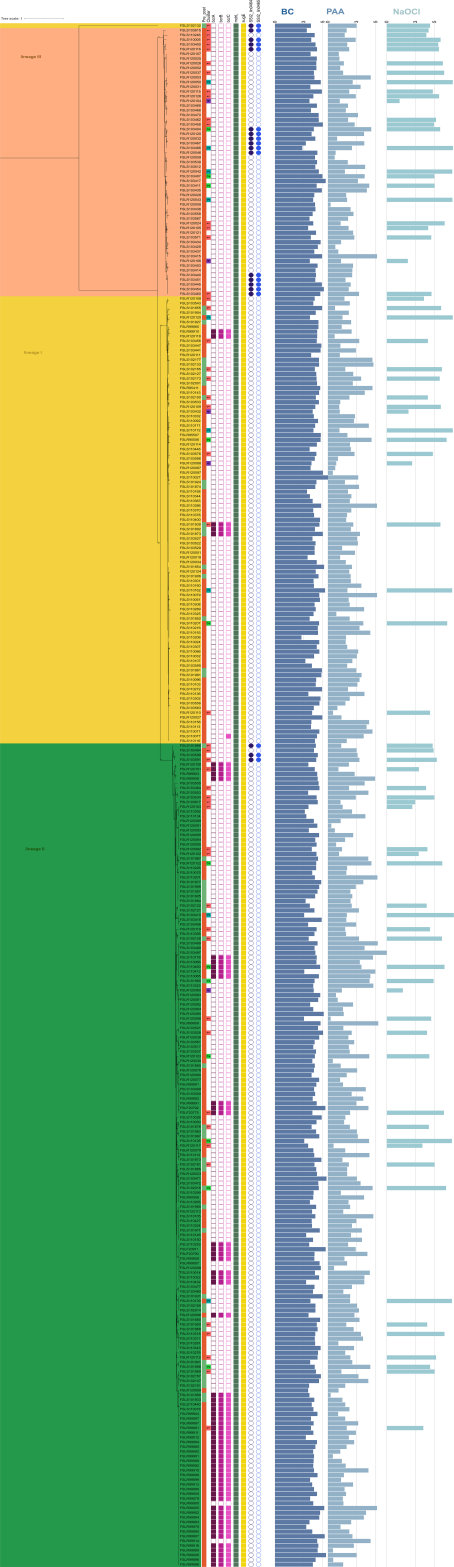
Maximum-likelihood phylogeny of 328 *L*. *monocytogenes* genomes constructed in RAxML v. 8.2.12 using core SNPs identified by kSNP3 as input; 1,000 bootstrap replicates were performed. Phylogeny is midpoint rooted, bootstrap values >80% are provided, and the scale in the top left corner indicates genetic distance. Branches are color-coded, showing isolates belonging to lineage I in yellow, lineage II in green, and lineage III in orange. The origin of isolates is provided in the Pre_post column, with green and red squares indicating isolates obtained from preharvest and postharvest sources, respectively. The Cluster column shows cluster assignments for isolates subjected to qtclust analysis of sanitizer log reduction data. Presence or absence of select sanitizer resistance genes/gene loci, including *bcrA*, *bcrB*, *bcrC*, *mdrL*, *sugE*, and SSI-2, is indicated by filled or unfilled squares/circles, respectively. Mean log reduction of a given isolate after treatment with benzalkonium chloride (BC), peroxyacetic acid (PAA), and sodium hypochlorite (NaOCl) is indicated by horizontal bar charts.

**Fig 3:**
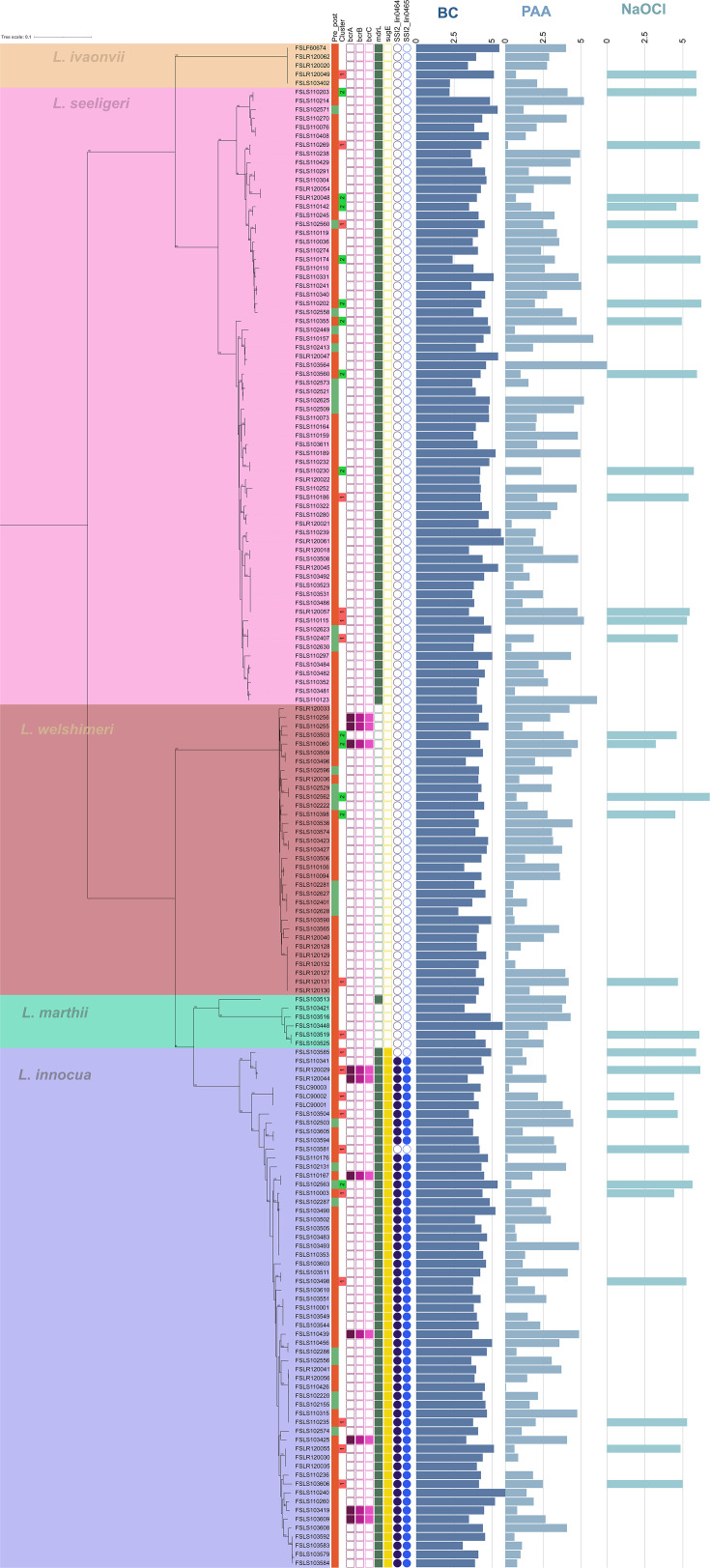
Maximum-likelihood phylogeny of 173 *Listeria* species genomes constructed in RAxML v. 8.2.12 using core SNPs identified by kSNP3 as input with 1,000 bootstrap replicates. Phylogeny is midpoint rooted, bootstrap values >80% are provided, and the scale in the top left corner indicates genetic distance. Branches are color-coded showing isolates belonging to *L. ivanovii* in orange, *L. seeligeri* in pink, *L. welshimeri* in red, *L. marthii* in green, and *L. innocua* in blue. The origin of isolates is provided in the Pre_post column, with green and red squares indicating isolates obtained from preharvest and postharvest sources, respectively. The Cluster column shows cluster assignments for isolates subjected to qtclust analysis of sanitizer log reduction data. Presence or absence of select sanitizer resistance genes/gene loci, including *bcrA*, *bcrB*, *bcrC*, *mdrL*, *sugE*, and SSI-2, is indicated by filled or unfilled squares/circles, respectively. Mean log reduction of a given isolate after treatment with benzalkonium chloride (BC), peroxyacetic acid (PAA), and sodium hypochlorite (NaOCl) is indicated by horizontal bar charts.

After exposure to BC, log reductions among the 501 isolates ranged from 2.76 log (FSL S11-0206, *L. monocytogenes*) to 5.73 log (FSL S10-3481, *L. seeligeri*), with a mean log reduction of 4.23 and standard deviation of 0.74 log (Fig. 1A to 3). No significant differences in log reductions were observed (i) between *L. monocytogenes* and *L*. spp. (estimated mean reductions of 4.21 log and 4.27 log, respectively [*P*=0.20]), (ii) between pre- and post-harvest isolates (estimated mean reductions of 4.26 log and 4.23 log, respectively [*P*=0.58]), (iii) across all six species (estimated mean reductions of 4.39 log, 4.23 log, 4.21 log, 4.16 log, 4.08 log, and 4.05 log for *L. seeligeri*, *L. innocua*, *L. monocytogenes*, *L. welshimeri*, *L. marthii*, and *L. ivanovii*, respectively [*P*=0.10]), or (iv) across the three *L. monocytogenes* lineages (estimated mean reductions of 4.27 log, 4.20 log, and 4.06 log for lineages I, II, and III, respectively [*P*=0.20]) (Tables 1 and 2).

**TABLE 1:** Linear regression models assessing the effects of (i) species of *Listeria*, (ii) *L. monocytogenes* vs. *L*. spp., (iii) preharvest vs. postharvest isolation source, and (iv) *L. monocytogenes* lineage on log reductions following exposure to each of the three sanitizers

Sanitizer^*a*^	Fixed effect	Df^*b*^	Sum Sq^*c*^	Mean Sq^*d*^	F value	Pr (>F)^*e*^
BC	Species	5	5.18	1.03	1.88	0.10
	Mono.spp^*f*^	1	0.91	0.91	1.65	0.20
	Prepost^*g*^	1	0.17	0.17	0.30	0.58
	Lineage	2	1.82	0.91	1.60	0.20
PAA	Species	5	127.3	25.45	9.53	<0.001
	Mono.spp^*f*^	1	40.90	40.86	14.95	<0.001
	Prepost^*g*^	1	3.70	3.73	1.35	0.25
	Lineage	2	6.76	3.38	1.23	0.29
NaOCl	Species	5	14.62	2.92	1.50	0.19
	Mono.spp^*f*^	1	6.88	6.88	3.53	0.06
	Prepost^*g*^	1	0.64	0.64	0.32	0.57
	Lineage	2	0.85	0.42	0.18	0.83

^*a*^ BC, benzalkonium chloride; PAA, peroxyacetic acid; NaOCl, sodium hypochlorite (exposures were performed at 300, 80, and 500 ppm, respectively).

^*b*^ Df, degrees of freedom.

^*c*^ Sum sq, sum of squares.

^*d*^ Mean Sq, mean of squares.

^*e*^ Pr(>F), p-value associated with the observed F-statistic.

^*f*^ Mono.spp, variable with two levels, *L. monocytogenes* and *L*. spp.

^*g*^ Prepost, variable with two levels, preharvest and postharvest.

**TABLE 2:** Emmeans of *Listeria* log reductions after exposure to use-level sanitizer concentrations

Sanitizer^*a*^	Fixed effect	Level	Mean log reduction (emmean)^*b*^	SE^*c*^	lower.CL^*d*^	upper.CL^*e*^
BC	Species	*innocua*	4.23	0.07	4.09	4.36
		*ivanovii*	4.05	0.23	3.59	4.51
		*marthii*	4.08	0.21	3.67	4.48
		*monocytogenes*	4.21	0.03	4.16	4.27
		*seeligeri*	4.39	0.06	4.27	4.51
		*welshimeri*	4.16	0.09	3.98	4.34
	Mono.spp^*f*^	*monocytogenes*	4.21	0.03	4.16	4.27
		Species	4.27	0.04	4.20	4.35
	Prepost^*g*^	Preharvest	4.23	0.03	4.18	4.28
		Postharvest	4.26	0.06	4.15	4.37
	Lineage	I	4.27	0.05	4.16	4.38
		II	4.20	0.04	4.13	4.27
		III	4.06	0.11	3.83	4.28
PAA	Species	*innocua*	2.38	0.14	2.11	2.66
		*ivanovii*	1.69	0.50	0.72	2.66
		*marthii*	2.11	0.44	1.23	2.97
		*monocytogenes*	2.46	0.06	2.35	2.57
		*seeligeri*	3.11	0.12	2.87	3.36
		*welshimeri*	3.39	0.19	3.02	3.76
	Mono.spp^*f*^	*monocytogenes*	2.46	0.06	2.34	2.57
		Species	2.85	0.08	2.69	3.01
	Prepost^*g*^	Preharvest	2.56	0.05	2.46	2.67
		Postharvest	2.71	0.11	2.49	2.93
	Lineage	I	2.46	0.11	2.25	2.68
		II	2.49	0.07	2.35	2.63
		III	2.11	0.23	1.65	2.56
NaOCl	Species	*innocua*	5.16	0.03	4.59	5.73
		*ivanovii*	5.89	0.99	3.94	7.83
		*marthii*	6.09	0.99	4.14	8.03
		*monocytogenes*	4.65	0.11	4.72	5.17
		*seeligeri*	5.59	0.26	5.07	6.11
		*welshimeri*	4.75	0.44	3.88	5.62
	Mono.spp^*f*^	*monocytogenes*	4.95	0.11	4.72	5.17
		Species	5.33	0.17	4.99	5.68
	Prepost^*g*^	Preharvest	5.24	0.11	4.83	5.24
		Postharvest	5.18	0.22	4.74	5.61
	Lineage	I	5.08	0.26	4.57	5.58
		II	4.91	0.16	4.60	5.23
		III	4.88	0.30	4.28	5.47

^*a*^ BC, benzalkonium chloride; PAA, peroxyacetic acid; NaOCl, sodium hypochlorite (exposures were performed at 300, 80, and 500 ppm, respectively).

^*b*^ Emmean, estimated marginal means.

^*c*^ SE, standard error.

^*d*^ lower.CL, lower confidence interval at 0.95.

^*e*^ upper.CL, upper confidence interval at 0.95.

^*f*^ Mono.spp, variable with two levels, *L. monocytogenes* and *L. *spp.

^*g*^ Prepost, variable with two levels, preharvest and postharvest.

After exposure to PAA, log reductions among the 501 isolates ranged from 0.15 (FSL S10-3565, *L. welshimeri*) to 6.16 log (FSL S10-3497, *L. monocytogenes*) with a mean log reduction of 2.59 and a standard deviation of 1.66 log (Fig. 1B to 3). Overall, *L*. spp. showed significantly lower log reductions to PAA compared to *L. monocytogenes* (estimated mean reductions of 2.46 log and 2.85 log, respectively [*P*<0.001]), indicating that *L*. spp. show reduced susceptibility to PAA (Tables 1 and 2). In addition, when comparing log reductions to PAA across all six species, *L. ivanovii* showed significantly lower log reductions compared to *L. welshimeri* (estimated mean reductions of 1.69 log and 3.39 log, respectively [*P*<0.001]); no significant differences were observed across the remaining species (i.e., *L. innocua*, *L. marthii*, *L. monocytogenes*, and *L. seeligeri* [*P*>0.05]). Additionally, no significant differences in log reductions were observed between pre- and post-harvest isolates (estimated mean reductions of 2.71 log and 2.56 log, respectively [*P*=0.25]) or across the three *L. monocytogenes* lineages (estimated mean reductions of 2.46 log, 2.49 log, and 2.11 log for lineages I, II, and III, respectively [*P* = 0.29]).

After exposure to NaOCl, log reductions among the 108 isolates ranged from 1.34 (FSL R12-0104, *L. monocytogenes*) to 7.02 log (FSL S10-3410, *L. monocytogenes*) with a mean log reduction of 5.06 and a standard deviation of 1.40 log (Fig. 1C to 3). No significant differences in log reductions were observed (i) between *L. monocytogenes* and *L*. spp. (estimated mean reductions of 4.95 log and 5.33 log, respectively [*P*=0.06]), (ii) between pre- and post-harvest isolates (estimated mean reductions of 5.24 log and 5.18 log, respectively [*P*=0.57]), (iii) across all six species (estimated mean reductions of 6.09 log, 5.89 log, 5.59 log, 6.16 log, 4.75 log, and 4.65 log for *L. marthii*, *L. ivanovii*, *L. seeligeri*, *L. innocua*, *L. welshimeri*, and *L. monocytogenes*, respectively [*P*=0.19]), or (iv) across the three *L. monocytogenes* lineages (estimated mean reductions of 5.08 log, 4.91 log, and 4.88 log for lineages I, II, and III, respectively [*P*=0.83]) (Tables 1 and 2).

Overall, isolates exhibited varying log reductions in response to all three sanitizers; the range of responses was larger for PAA and NaOCl than for BC. A generalized linear model estimated the variances for the subset of 108 isolates that were characterized with all three sanitizers to be 1.12, 1.00, and 0.51 for PAA, NaOCl, and BC, respectively; these variances differed significantly (Bartlett test, *P*<0.0001).

### Cluster analysis identified one cluster of five *L. monocytogenes* that showed reduced susceptibility to all three sanitizers

**Fig 4:**
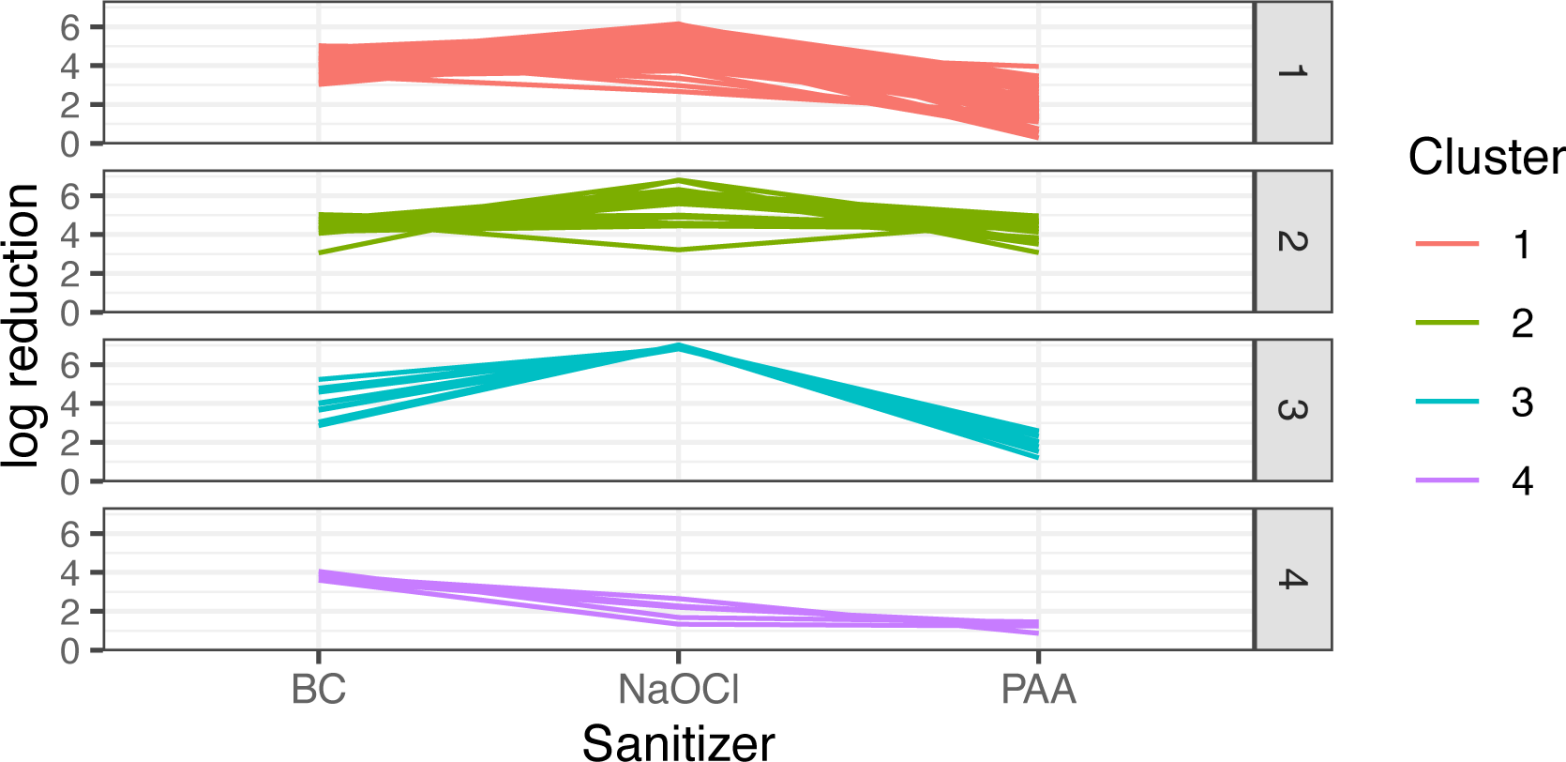
In total, 108 *Listeria* isolates were grouped into four clusters according to their log reductions when exposed to benzalkonium chloride (BC), sodium hypochlorite (NaOCl), and peroxyacetic acid (PAA) using quality threshold clustering (QTC). Clusters 1, 2, 3, and 4 are represented by 69, 25, 9, and 5 isolates, respectively.

To determine whether isolates represented distinct groups based on log reduction data, qtclust (QTC) analysis was performed on the 108 isolates for which log reduction data were available for all three sanitizers. This analysis identified four clusters (Fig. 2 and 3). Clusters 1 and 2 included both *L. monocytogenes* (50 and 12 isolates, respectively) and *L*. spp. (19 and 13 isolates, respectively), whereas clusters 3 and 4 included exclusively *L. monocytogenes* (nine and five isolates, respectively). Isolates in cluster 1 showed higher log reductions for BC and NaOCl (mean of 4.15 log and 4.92 log, respectively) compared to PAA (mean of 2.15 log), whereas isolates in cluster 2 showed relatively high log reductions for all sanitizers (mean of 4.42 log, 5.48 log, and 4.35 log for BC, NaOCl, and PAA, respectively) (Fig. 4). Isolates in cluster 3 showed (i) high log reductions for NaOCl (mean of 6.89 log), (ii) lower log reductions than clusters 1 and 2 for BC (mean of 3.8 log), and (iii) low log reductions for PAA (mean of 1.99 log). All isolates in cluster 4 exhibited relatively low log reductions across all three sanitizers (mean of 3.81 log, 2.03 log, and 1.23 log for BC, NaOCl, and PAA, respectively). The isolates from cluster 4 represented all three *L. monocytogenes* lineages, including FSL R12-0088 (lineage I), FSL S10-3432 (lineage I), FSL R12-0060 (lineage II), FSL R12-0104 (lineage III), and FSL R12-0106 (lineage III) (Fig. 2).

### Quat resistance genes were significantly overrepresented in *L. monocytogenes* isolates

**Fig 5:**
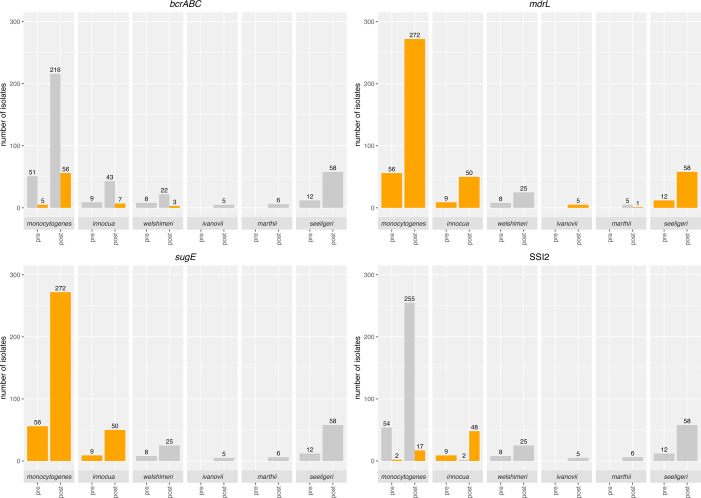
Presence and absence of sanitizer resistance genes (i.e., *bcrABC*, *mdrL*, *sugE1/2*, and SSI-2) in 501 *Listeria* isolates. Bars in orange indicate gene presence, and bars in gray indicate gene absence; numbers above each bar indicate the number of isolates. For species where all isolates show either the presence or absence of a given gene, only a single bar is shown. Pre- and post-labels represent isolates obtained from preharvest and postharvest environments, respectively. All isolates classified as positive for a given gene showed full-length *bcrABC*, *sugE1/2*, and SSI-2, except for one isolate (FSL S11-0077) that showed the presence of *bcrC* with >90% sequence identity and query coverage but did not show the presence of *bcrB* and *bcrA* (this isolate was classified as not showing the presence of *bcrABC*).

Of the six loci that encode for efflux pumps known to confer tolerance to quats that were queried for here (i.e., *bcrABC*, *mdrL*, *emrE*, *qacA, qacE*, *qacH*, and *sugE1/2*), only *bcrABC, mdrL,* and *sugE1/2* were represented among the 501 *Listeria* isolates (*n* = 70, *n* = 463, and *n* = 387, respectively; see Fig. 5). The *bcrABC* operon was significantly overrepresented among *L. monocytogenes* isolates (60/328 [18.3%]) compared to *L*. spp. (10/173 [5.8%]) (*P*<0.001). For *L. monocytogenes*, *bcrABC* was identified in both preharvest (5/56 [8.9%]) and postharvest (55/272 [20.2%]) isolates, whereas for *L*. spp., *bcrABC* was only identified in postharvest isolates representing *L. innocua* (7/59 [11.9%]) and *L. welshimeri* (3/33 [9.0%]); to our knowledge, these are the only *L*. spp. that have previously been described to carry *bcrABC* (30–32). Across all species, *bcrABC* was significantly more likely to be present in postharvest (65/416 [15.6%]) than in preharvest isolates (5/85 [5.9%]) (*P*<0.05) (Table 3). *mdrL* was present in all *L. monocytogenes, L. innocua, L. ivanovii,* and *L. seeligeri*, as well as one *L. marthii* isolate (Fig. 5). The *sugE* operon (comprised of both *sugE1* and *sugE2*) was identified in all *L. monocytogenes* and *L. innocua* isolates, but was not found in the 114 isolates representing all other species (i.e., *L. ivanovii*, *L. marthii*, *L. seeligeri*, and *L. welshimeri*). Additionally, the stress survival islet SSI-2, which has been associated with oxidative stress tolerance and thus may confer reduced susceptibility to oxidizing sanitizers (33), was significantly more common in *L. innocua* isolates (57/59 [96.6%]) compared to isolates representing all other *Listeria* species (*P*<0.001) (Table 3). In addition to *L. innocua*, *L. monocytogenes* was the only other species where SSI-2 was identified (19/328 isolates [5.8%]) (Fig. 5).

**TABLE 3:** Model parameters for linear regression models for the presence and absence of sanitizer resistance genes among the 501 *Listeria* isolates

Resistance gene	Fixed effect	Df^*a*^	Sum sq^*b*^	Mean Sq^*c*^	F value	Pr (>F)^*d*^
*bcrABC*	Prepost^*e*^	1	0.67	0.67	5.76	0.017
	Species	5	2.34	0.47	4.02	0.001
	Species*prepost^*f*^	3	0.12	0.04	0.35	0.783
*mdrL*	Prepost^*e*^	1	0.03	0.03	20.13	<0.001
	Species	5	34.25	6.85	4036.05	<0.001
	Species*prepost^*f*^	3	0.00	0.00	0.00	1
*sugE*	Prepost^*e*^	1	0.00	0.00	0.00	<0.001
	Species	5	88.05	17.61	0.00	<0.001
	Species*prepost^*f*^	3	0.00	0.00	0.00	0.755
SSI2	Prepost^*e*^	1	0.05	0.05	1.26	0.26
	Species	5	44.60	8.92	221.36	<0.001
	Species*prepost^*f*^	3	0.03	0.01	0.27	0.845

^*a*^ Df, degrees of freedom.

^*b*^ Sum sq, sum of squares.

^*c*^ Mean Sq, mean of squares.

^*d*^ Pr(>F), significance probability associated with F value.

^*e*^ Prepost, variable with two levels, preharvest and postharvest.

^*f*^ The * indicates the interaction effect between species and prepost variables.

Linear regression analysis did not find any evidence that the presence/absence of the three known quat resistance genes/gene loci identified among *Listeria* isolates here (i.e., *bcrABC*, *mdrL*, and *sugE1/2*) were significantly associated with *Listeria* log reductions to BC (*P* = 0.26, *P* = 0.33, and *P* = 0.13, respectively; Table 4). Similarly, regression analysis did not find any evidence for statistically significant associations between the presence/absence of SSI-2 and *Listeria* log reductions when the isolates were exposed to PAA (*P* = 0.19) or NaOCl (*P* = 0.63).

**TABLE 4:** Linear model parameters for associations between *Listeria* sanitizer resistance genes and log reductions for the three sanitizers

Sanitizer^*a*^	Fixed effect^*b*^	Estimate	Standard error	*t*-value^*c*^	*P* value
BC	*bcrABC*	−0.07	0.07	−1.13	0.26
	*mdrL*	0.09	0.09	0.98	0.33
	*sugE*	−0.08	0.05	−1.52	0.13
PAA	SSI2	−0.18	0.14	−1.31	0.19
NaOCl	SSI2	0.12	0.25	0.49	0.63

^*a*^ BC, benzalkonium chloride; PAA, peroxyacetic acid; NaOCl, sodium hypochlorite (exposures were performed at 300, 80, and 500 ppm, respectively).

^*b*^ Only associations between sanitizer log reductions and presence of genes previously linked to reduced tolerance to a given sanitizer were tested.

^*c*^ *t*-value indicates the number of standard errors by which the estimated effect differs from zero.

### Postharvest isolates are significantly more likely to carry a plasmid compared to preharvest isolates

**Fig 6:**
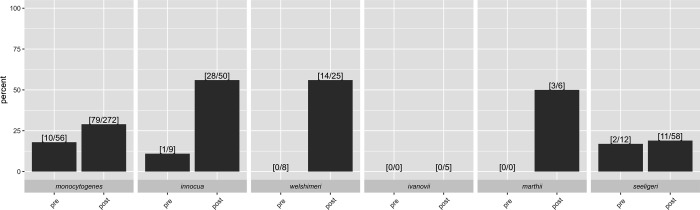
Bar graphs indicating the percentage of isolates that carry a plasmid as identified through PLATON for *L. monocytogenes, L. innocua, L. welshimeri, L. ivanovii, L. marthii,* and *L. seeligeri*. Bars above pre- and post-labels represent the percentage of isolates that show evidence for the presence of a plasmid and were obtained from preharvest and postharvest environments, respectively. Fractions at the top of each bar indicate the number of isolates that show evidence for presence of a plasmid/total number of isolates represented for a given species and environment (pre- and post-harvest).

Plasmidome analysis identified at least one likely plasmid in 29.3% (147/501) of *Listeria* isolates; for six of these isolates (four *L. monocytogenes* and two *L. innocua*), two distinct *oriR* were identified, indicating the likely presence of two plasmids (Fig. 6). *L. innocua* isolates (29/59 [49.2%]) were significantly more likely to carry a plasmid than *L. monocytogenes* (89/328 [27.1%]), *L. ivanovii* (0/5 [0%]), and *L. seeligeri* (13/70 [18.6%]) (*P*<0.01) (Table 5). The *bcrABC* operon was identified as encoded on a plasmid for 64 of the 70 *Listeria* isolates, as supported by the identification of *bcrABC* on a contig that was also classified as a plasmid by PLATON. For the remaining six isolates, PLATON was unable to determine if the contig that carried *bcrABC* was present on a plasmid. This may be due to the inability of the program to detect the necessary plasmid markers or the lack of sufficient sequence data in the relevant regions to confidently classify the contigs as plasmid-borne.

**TABLE 5:** Logistic regression results assessing associations between *Listeria* isolate characteristics and plasmid presence

Model	Fixed effect	Estimate	Standard error	*t*-value^*a*^	*P* value
	(Intercept)	0.56	0.06	8.97	<0.001
Prepost^*b*^ (reference: postharvest)	Preharvest	−0.45	0.16	−2.81	<0.01
Species (reference: *innocua*)	*ivanovii*	−0.56	0.21	−2.70	<0.01
	*marthii*	−0.06	0.19	−0.31	0.75
	*monocytogenes*	−0.27	0.07	−3.97	<0.001
	*seeligeri*	−0.37	0.09	−4.35	<0.001
	*welshimeri*	0.00	0.11	0.00	1
Prepost*species^*c*^ (reference: postharvest*innocua)	Preharvest: *ivanovii*	–^*d*^	–	–	–
	Preharvest: *marthii*	–	–	–	–
	Preharvest: *monocytogenes*	0.34	0.17	1.95	0.05
	Preharvest: *seeligeri*	0.43	0.21	2.00	<0.05
	Preharvest: *welshimeri*	−0.11	0.24	−0.46	0.64

^*a*^ *t*-value indicates the number of standard errors by which the estimated effect differs from zero.

^*b*^ Prepost, variable with two levels, preharvest and postharvest.

^*c*^ * indicates the interaction effect between prepost and species variables.

^*d*^ –, data not available as no *L. ivanovii* or *L. marthii *isolates in the data set were obtained from preharvest environments.

### *Listeria* inactivation to sanitizers was significantly associated with SNPs in core genes

In addition to queries for genes/loci with known association to sanitizer tolerance phenotypes, GWAS analyses were performed to screen for genes or SNPs that might be associated with *Listeria* log reductions to each of the three sanitizers. For these analyses, *Listeria* genomes were divided into smaller subpopulations to overcome potential issues with genome plasticity (e.g., increased diversity in gene content and sequence diversity with increased number of isolates that are not closely related [34]). Specifically, GWAS analyses with log reduction data for BC and PAA were conducted for subpopulations representing (i) *L*. spp. (i.e., all *Listeria* species excluding *L. monocytogenes*), (ii) *L. monocytogenes*, (iii) *L. monocytogenes* lineage I, (iv) *L. monocytogenes* lineage II, (v) *L. monocytogenes* lineage III, (vi) *L. innocua*, (vii) *L. seeligeri*, and (viii) *L. welshimeri* (Table 6). For NaOCl, GWAS analyses were conducted for subpopulations of (i) *L*. spp., (ii) *L. monocytogenes*, and (iii) *L. monocytogenes* lineage II.

**TABLE 6:** Number of pangenome genes included in treeWAS analyses for different *Listeria* subpopulations

*Listeria* subpopulation	No. of isolates	No. of pangenome genes
Subpopulations used for treeWAS analyses of BC and PAA log reductions		
*L. monocytogenes*	328	6,285
*L*. spp.	173	8,291
*L. seeligeri*	70	4,892
*L. innocua*	59	4,780
*L. welshimeri*	33	4,172
*L. monocytogenes* lineage I	170	4,339
*L. monocytogenes* lineage II	210	5,397
*L. monocytogenes* lineage III	22	3,933
Subpopulations used for treeWAS analyses of NaOCl log reductions		
*L. monocytogenes*	76	5,536
*L*. spp.	32	6,173
*L. monocytogenes* lineage II	45	4,794

None of the GWAS performed across each combination of subpopulation and sanitizer type yielded a significant association between genes present in each subpopulation’s pangenome and phenotypic log reduction data. However, GWAS identified several SNPs in core genes in select *Listeria* subpopulations that were significantly associated with log reductions to BC, PAA, and NaOCl. For BC, we identified 22 SNPs, represented across a total of 10 genes, that were significantly associated with BC log reduction values; SNPs were identified across subpopulations representing *L. monocytogenes* lineage III (13 SNPs), *L*. spp. (3 SNPs), *L. innocua* (2 SNPs), and *L. seeligeri* (4 SNPs) (Table S2). Of the 10 genes identified here, which showed the presence of significant SNPs, only SNPs in *yedJ* were detected across multiple subpopulations (i.e., *L. spp*. and *L. seeligeri*).

For PAA, 117 SNPs, represented in a total of 61 genes, were significantly associated with PAA log reduction values; SNPs were identified across subpopulations representing *L. monocytogenes* (3 SNPs)*, L. monocytogenes* lineage I (4 SNPs), II (5 SNPs), and III (101 SNPs); *L*. spp. (1 SNP); and *L. innocua* (3 SNPs) (Table S2). Of the 61 genes identified here that showed the presence of significant SNPs, only SNPs in *mngB* were detected across multiple subpopulations (i.e., *L. monocytogenes*, *L. monocytogenes* lineage II, and *L. innocua*).

For NaOCl, 105 SNPs, represented in a total of 21 genes, were significantly associated with NaOCl log reduction values; SNPs were identified across subpopulations representing *L. monocytogenes* (22 SNPs)*, L. monocytogenes* lineage II (81 SNPs), and *L*. spp. (2 SNPs) (Table S2). Of the 21 genes in which significant SNPs were identified, SNPs in *cycA*, *cycD*, *gmuC*, and *ptsI* were each detected across two subpopulations (i.e., *L. monocytogenes* and *L. monocytogenes* lineage II).

## DISCUSSION

Concerns have been raised that *Listeria* develops reduced susceptibility to sanitizers used in food packing/processing environments, which could facilitate persistence and increase the risk of finished product contamination. Information on *Listeria* susceptibility to sanitizers at use-level concentrations, especially for isolates associated with the fresh produce environments, is lacking. Here, we collected WGS data, as well as data on sanitizer susceptibility, for 501 produce-associated *Listeria* isolates. Overall, our data showed that (i) across a large and diverse collection of *Listeria* isolates, log reductions following exposure to use-level concentrations of BC, PAA, and NaOCl can vary widely; (ii) reduced sanitizer susceptibility is uncommon and not linked to the presence of several previously reported sanitizer resistance genes/loci (e.g., *bcrABC*, *mdrL*, and SSI-2); and (iii) WGS appears to be of limited value in predicting the potential for *Listeria* to show reduced susceptibility to use-level sanitizer concentrations.

### *Listeria* isolates show a wide range of sanitizer susceptibility, highlighting the need to consider log reduction variability in addition to mean log reduction data for sanitizer comparisons

Here, we provide a large data set on the log reductions of 501 produce-associated *Listeria* isolates from the U.S. after exposure to three sanitizers commonly used for sanitation in food packing/processing environments. Overall, our data showed that under the specific conditions used (e.g., pre-growth condition, exposure time, and sanitizer concentration), exposure to NaOCl yielded the highest mean log reduction of *Listeria* (5.06 log), followed by BC (4.23 log) and PAA (2.59 log). Importantly, our data showed that variability in log reductions differed significantly across all three sanitizers, with exposure to BC resulting in the smallest variance in log reductions (0.51), followed by NaOCl (1.00) and PAA (1.12). Bridier et al. (35) made similar observations when exposing 10 strains of *L. monocytogenes* to PAA and BC; they specifically observed that the variance for log reductions to PAA was significantly larger than that for BC.

The larger variance of log reductions for NaOCl and PAA, compared to BC, could be due to genetic variability among isolates, which could impact the types of stress response mechanisms employed following exposure to sanitizers with different modes of action. For example, as the primary mode of action of BC is to destabilize the cell membranes through electrostatic attraction (36), only a handful of stress response mechanisms that target the cell membrane specifically (e.g., expression of efflux pumps (37)) can be used to mitigate cell death. On the other hand, as PAA and NaOCl can facilitate cell death via nonselective oxidation of various enzymes and other cellular components required for cell metabolism (38, 39), *Listeria* can similarly employ a variety of stress response mechanisms, which can vary greatly across isolates, to repair protein/nucleic acid damage and mitigate cell death (40). The variability in log reductions observed here could also be impacted by differences in the ability of different isolates to enter an injured or viable but non-culturable (VBNC) state (41, 42), which could be particularly relevant for NaOCl and PAA, as both of these sanitizers have been reported to induce a VBNC state in *Listeria* (42–44). For example, in Truchado et al. (44), the authors reported that, while exposure to 10 ppm sodium hypochlorite yielded an estimated 99.9% reduction of *L. monocytogenes* culturable cells, only 65.7% of cells were confirmed dead, whereas 27.4% were injured and viable, and 6.9% were VBNC. Similarly, two studies (42, 43) reported that exposure of *L. monocytogenes* to ≥30 ppm PAA for 30 s to 1 min resulted in high log reductions (4–5 log) via culture-dependent enumeration on selective agar, but low log reductions (1–2 log) via culture-independent enumeration (e.g., PMA-qPCR, and EMA + PMA qPCR), indicating that PAA (i) has limited ability to compromise cell membrane integrity and (ii) may potentially play a role in the induction of a VBNC state in *L. monocytogenes*. Thus, varying degrees of cellular injury due to within-strain and between-strain variability are likely contributing factors for an overall larger range of log reductions observed within a population following exposure to NaOCl or PAA (43–45). More broadly, while our experiments were conducted under standardized conditions, it is expected that environmental and experimental conditions (e.g., conditions under which sanitizer exposure assays are performed) may yield higher variability with some sanitizers than others (46). Variability in log reductions for a given population will also increase if strains are pre-grown under different conditions prior to sanitizer exposure. For instance, when five *Listeria* strains were pre-grown under seven different conditions prior to PAA exposure, the variance component for “pre-growth condition” was more than eight times larger than the variance component for “strain” (45).

Overall, our findings, along with previous studies, highlight the importance of evaluating the variability of log reductions, in addition to mean log reduction, as well as sanitizer-specific (e.g., sanitizer mode of action) and strain-specific (e.g., stress tolerance) factors that can contribute to variation in *Listeria* inactivation when interpreting data from sanitizer inactivation assessments. These same factors can also be considered in trade-off risk assessments. For example, the risk of *Listeria* surviving exposure to PAA in produce packinghouses/processing facilities may be higher than other sanitizers (e.g., BC), given that exposure to use-level concentrations of PAA (i) yielded the largest variance of log reductions among produce-associated *Listeria* isolates evaluated here and (ii) has been shown to induce a VBNC state in *L. monocytogenes* (42). Including this and other relevant data in trade-off risk assessments can aid in informing appropriate selection of sanitizers for food facility sanitization and support additional decision-making related to *Listeria* control in food processing/packing environments.

We also assessed whether *Listeria* susceptibility to use-level sanitizer concentrations might vary for populations representing different species and isolation sources. In general, our data showed limited effects of the species of *Listeria* on sanitizer sensitivity. We only found a single significant association (*L*. spp. was found to be significantly less susceptible to PAA than *L. monocytogenes*), which supports the value of using *L*. spp. as index organisms for the presence of *L. monocytogenes* in food processing environments as part of environmental monitoring programs (47). We also found no significant effect of different isolation sources (pre- or post-harvest) on sanitizer susceptibility. It is possible that isolates may be exposed to stressors in the preharvest environment that are similar to the stress caused by exposure to sanitizers in the postharvest environment. For example, *Listeria* could potentially encounter oxidative stress, the main mechanism of action of PAA and NaOCl (38, 39), in the preharvest environment from (i) pesticides or herbicides, (ii) H_2_O_2_ formation at the anoxic interface of the soil, or (iii) UV radiation through sunlight (48, 49). Overall, while we observed substantial variability in *Listeria* log reductions to PAA and NaOCl, and to a lesser extent BC, our data indicate that this variability is not primarily driven by factors such as species or isolation source. Thus, although future studies that include additional isolates representing a wider diversity of isolation sources and subtypes may be useful, ensuring a large sample size (i.e., inclusion of a large number of isolates) in sanitizer susceptibility assays is likely more critical for capturing the full range of phenotypic responses.

### Reduced susceptibility of *Listeria* to use-level sanitizer concentrations is not linked to the presence of several known sanitizer resistance genes

Previous studies have identified several genes/loci associated with *Listeria* tolerance to low levels of quat-based sanitizers, including *bcrABC* (20), *emrE* (18), *mdrL* (16), *qacH* (19), and *sugE1/2* (50). For example, a recent large-scale disinfectant sensitivity study observed that, among 388 *L*. *monocytogenes* isolates classified as BC tolerant based on an MIC cutoff of ≥1.25 ppm, 95% (*n* = 368) harbored one of four quat-resistance genes (i.e., *bcrABC*, *qacH*, *emrC*, or *emrE* (51)). While our study identified *bcrABC*, *mdrL*, and *sugE1/2* in a number of isolates (70, 463, and 387, respectively), none of these genes/loci were significantly associated with reduced susceptibility of *Listeria* to a use-level concentration (i.e., 300 ppm) of BC. Importantly, Bolten et al. (22) reported that log reductions of *Listeria* following exposure to 300 ppm BC were not significantly different when comparing strains that carried *bcrABC* or *qacH* (mean reduction of 4.47 log) vs. strains that did not carry a quat-resistance gene (mean reduction of 4.57 log). These data suggest that known quat-resistance genes/loci have limited impact on reducing *Listeria* susceptibility to use-level BC concentrations and, comparatively, may only confer a selective survival advantage at substantially lower quat concentrations (e.g., 50×–100× dilution) than those recommended for application in food processing environments. This hypothesis is supported by previous reports indicating that known quat-resistance genes/loci typically only confer a 2-fold to 4-fold increase in *Listeria* tolerance to quats (13, 22, 50, 52, 53). For example, Xu et al. (52) observed that a *L. monocytogenes* strain carrying a *bcrABC*-containing plasmid had a 2-fold higher BC minimum inhibitory concentration (MIC) (MIC: 28 ppm), compared to an isogenic plasmid-cured strain (MIC: 14 ppm). Similarly, Jiang et al. (50) observed a 2-fold higher BC MIC in a wild-type *L. monocytogenes* strain carrying *sugE1* and *sugE2* (MIC of 8 ppm), compared to its double deletion mutant *ΔsugE1ΔsugE2* (MIC of 4 ppm). Trends across additional studies that characterized *Listeria* quat tolerance phenotypes among food-associated isolates further support this pattern (13, 53). Moreover, Bolten et al. (22) and Kastbjerg and Gram (54) showed that, while incremental adaptation experiments were able to yield *Listeria* isolates with increased tolerance to quat concentrations 2-fold to 8-fold higher than their un-adapted wild-type counterparts, both adapted isolates and wild-type strains remained similarly susceptible to quats at use-level concentrations. Collectively, these findings indicate that mechanisms conferring low-level tolerance to quats have limited impact on reducing *Listeria* susceptibility to use-level quat concentrations.

Although it is tempting to speculate that the presence of genetic loci linked to oxidative stress resistance (e.g., SSI-2) would reduce *Listeria* susceptibility to use-level concentrations of PAA or NaOCl, no such association was found here. While previous data in isogenic mutants have shown reduced susceptibility of a SSI-2 null mutant to 10 mM cumene hydroperoxide (33), we are not aware of data on the susceptibility of SSI-2 mutants to PAA or NaOCl. Moreover, previous efforts to identify specific genetic elements associated with *Listeria* tolerance to PAA and NaOCl have yielded limited findings (51, 55, 56). Nevertheless, transcriptomic evidence increasingly suggests that molecular mechanisms, such as transient activation of oxidative stress response genes, contribute to short-term tolerance or acclimation of *Listeria* to sublethal concentrations of oxidizing sanitizers (57, 58). Further research on these and other acclimation mechanisms may be valuable, including future studies evaluating how pre-adaption to low levels of PAA and NaOCl may influence *Listeria* susceptibility to use-level concentrations of these oxidizing sanitizers.

Interestingly, we found *bcrABC* to be more prevalent in postharvest than in preharvest isolates (15.6% and 5.9%, respectively), which could suggest that produce-associated *Listeria* obtained from postharvest environments are more likely to be exposed to environmental pressures that select for maintenance of *bcrABC* compared to isolates obtained from preharvest environments. For example, in postharvest environments (e.g., packinghouses and fresh-cut processing facilities), *Listeria* may be more frequently exposed to conditions that contribute to selection of low-level quat tolerance conferred by *bcrABC*, including instances where (i) quats are frequently used and (ii) exposure to diluted quat concentrations is likely (e.g., drains, P-traps, or other locations with standing water) (59). Prevalence of *bcrABC-*positive *Listeria* in postharvest environments may also be facilitated by the fact that *bcrABC* is often localized on a plasmid and thus can possibly be dispersed across different *Listeria* species and subtypes via horizontal gene transfer (30).

Overall, while our data suggest that the emergence of produce-associated *Listeria* with tolerance to use-level concentrations of BC, PAA, or NaOCl through the acquisition of known resistance genes is unlikely, the same cannot be said when considering exposure to low sanitizer concentrations. This further underscores the importance of ensuring appropriate dilution, application, and distribution of quat-based sanitizers throughout food packing/processing environments, to ensure effective sanitization and control of *Listeria*. In many cases, this may require equipment disassembly prior to cleaning and sanitation in order to assure that all components of a piece of equipment can be exposed to appropriate use-level concentrations of quats or other sanitizers (2).

### While GWAS supports a lack of links between gene presence/absence and sanitizer reduced susceptibility, it suggests that SNPs in stress response-associated genes should be further explored as markers for differences in reduced sanitizer susceptibility

In recent years, GWAS has become a valuable tool in comparative genomics to link phenotypes to key genetic markers and loci. For example, previous studies have utilized GWAS to identify genes/SNPs associated with antimicrobial resistance and virulence phenotypes in various bacterial pathogens (60–62). More specifically, one GWAS has been successfully applied to 51 *L*. *monocytogenes* isolates that represented lineage I and II and allowed the identification of SNPs, genes, and specific sub-lineages associated with cold growth (26). In the GWAS performed here, we did not identify specific genes associated with log reductions to the three different sanitizers. This is also consistent with the fact that our more targeted analyses discussed above did not identify any known quat-resistance genes as associated with BC log reductions. One possible explanation for these findings is that the phenotype of interest examined here (i.e., reduced susceptibility of *Listeria* to use-level sanitizer concentrations) might be highly polygenic (i.e., influenced by a combination of multiple genes/gene loci), which can interfere with the ability of GWAS to identify significant associations specific to individual genes (63). Importantly, it would not be unexpected for reduced susceptibility of *Listeria* to PAA and NaOCl to be polygenic as (i) oxidative sanitizers cause general cellular damage through oxidation of multiple targets (e.g., lipids, nucleic acid, and membrane protein) and (ii) oxidative stress resistance often utilizes multiple detoxification pathways (e.g., superoxide dismutase, glutathione peroxidase, and catalases) (57). A likely multigenic mechanism for reduced susceptibility to oxidative sanitizers in *Listeria* is also supported by Pleitner et al. (64), who identified 340 differentially expressed genes when *L. monocytogenes* 10,403s was exposed to the oxidizing agent chlorine dioxide. Further exploration into the possible mechanism of sanitizer adaptation, and particularly adaptation to oxidizing sanitizers, could thus be valuable and may need to use different approaches. For example, transposon insertion sequencing (Tn-seq) or similar mutagenesis approaches could be employed for select isolate groups to identify genes/loci that may contribute to the observed phenotypes (65).

Interestingly, we identified several SNPs in *Listeria* core genes that were significantly associated with log reductions to one of the three sanitizers. In some cases, these SNPs were identified in genes that may confer certain functions that play a role in tolerance to specific sanitizers. For example, *ybhS*, for which GWAS identified one SNP associated with log reductions to BC for the *L. monocytogenes* lineage III subpopulation, encodes an inner membrane protein component of YbhFSR, an ABC efflux pump that has been shown to confer BC tolerance in *Aliarcobacter butzleri* (66). Thus, it is possible that *ybhS* may play a similar role in BC tolerance in *L. monocytogenes*. Additionally, SNPs in several genes with homologies to phosphotransferase system (PTS) genes were also found to be significantly associated with PAA log reduction data. These included SNPs identified in (i) *licC* and *licH*, which have been reported to be part of a beta-glucoside utilization system in *Bacillus subtilis* (67), and (ii) *manX,* which has been reported to be part of the *E. coli manXYZ* operon that encodes a PTS transporter involved in transport of a number of different sugars, including mannose (68). Interestingly, impaired expression of a mannose PTS has previously been reported to lead to increased peroxide stress sensitivity in *Lacticaseibacillus plantarum* (69). In addition, another study showed that PTS-mediated glucose transport was induced under oxidative stress in *E. coli* (70). Together, these findings suggest that some PTS may be involved in conferring oxidative stress tolerance and thus may play a similar role in reducing the susceptibility of *Listeria* to PAA. While our findings imply that some SNPs in genes with previously reported links to sanitizer tolerance or oxidative stress response function may play a role in *Listeria* susceptibility to sanitizers, additional efforts are needed to confirm this, including experimental data on whether and how the SNPs identified here impact function and phenotypes.

### Conclusions

Our findings support that genetically mediated sanitizer tolerance of *sensu stricto Listeria* species may be of less importance than previously suggested. Specifically, data presented here suggest that the presence/absence of sanitizer resistance and stress response genes may only contribute to *Listeria* sanitizer tolerance at low-level concentrations, with limited impact on reducing *Listeria* susceptibility to industry-relevant use level concentrations. We also observed high variability in *Listeria* log reductions following exposure to certain sanitizers, most notably PAA. Given these findings, it may be prudent to focus criteria for sanitizer selection and decisions on the value of sanitizer rotation on factors outside those related to concerns regarding the emergence of sanitizer tolerance. However, our data suggest that further research on the possible impacts of SNPs, including those found here to be associated with enhanced sanitizer tolerance phenotypes, may be valuable to identify markers for differences in sanitizer susceptibility.

## MATERIALS AND METHODS

### Bacterial isolate selection and storage

Isolates representing six *sensu stricto Listeria* species, including *L. monocytogenes, L. innocua, L. ivanovii, L. marthii, L. seeligeri,* and *L. welshimeri*, were selected from a variety of sources associated with the production of fresh produce. Isolates represented both preharvest (e.g., soil and water) and postharvest (e.g., packinghouses and processing facilities) environmental sources in the continental United States; many isolates were collected as part of previous research studies (6, 45, 71–74) (Table 7).

**TABLE 7:** Sources of isolates included in the study

Source category	*L. monocytogenes* (no. of isolates)	*L*. spp. (no. of isolates)	Short description and reference^*a*^
Preharvest isolates (*n* = 85)			
I	15	29	Isolates obtained from spinach, soil, and water taken from a produce field in New York State, as described in Weller et al. (71)
II	41	0	Isolates obtained from spinach, soil, water, and drag swabs taken from a produce field in New York State, as described in Harrand et al. (72)
Postharvest isolates (*n* = 416)			
III	70	54	Isolates obtained from 11 produce packinghouses in the US, as detailed in Estrada et al. (73)
IV	78	58	Isolates obtained from three produce packinghouses and five fresh-cut processing facilities in the US, as detailed in Sullivan and Wiedmann (74)
V	23	22	Isolates obtained from a stone fruit packinghouse in Virginia, as detailed in Bardsley et al. (6)
VI	54	0	Isolates associated with high-profile produce outbreaks (e.g., cantaloupe, apple, sprouts, and salads), as described by Harrand et al. (45)
VII	4	10	Miscellaneous isolates from ready-to-eat (RTE) produce, such as fresh-cut salads, coleslaw, and bean/broccoli sprouts, available in the Cornell strain collection
VIII	43	0	Isolates obtained from onion, cantaloupe, and other produce packing environments and provided by other researchers

^*a*^ Detailed information associated with individual isolates can be obtained from the FoodMicrobeTracker database (https://www.foodmicrobetracker.net/login/login.aspx) by searching for a given isolate’s ID number (e.g., FSL C9-0001; see Table S1 for all isolate IDs).

All *Listeria* isolates were stored in brain heart infusion (BHI; BD Diagnostics, Sparks, MD) broth with 15% glycerol at −80°C. Isolates were streaked from the −80°C glycerol stocks onto BHI agar plates, and plates were incubated at 37°C for 24 h. After incubation, plates were stored at 4°C for at least 24 h but no longer than 7 days prior to experiments.

### Bacterial growth curves

Bacterial growth curves were generated for all isolates to estimate the time point at which the isolates reached early stationary phase (further referred to as t_Nmax_) when incubated at 22°C in BHI broth. For bacterial culture preparation, 5 mL of BHI broth was inoculated with a single colony, followed by static incubation at 22°C for approximately 40 h. Cultures were then diluted 1:1,000 to achieve approximately 10^5^ CFU/mL, and 300 μL of each diluted culture was transferred to a 96-well flat-bottom plate. The plates were incubated for 48 h at 22°C in a Synergy H1 Hybrid Multi-Mode Microplate Reader (BioTek, Winooski, VT), and absorbance was measured at OD_600_ every 10 min. The Growthcurver package v. 0.3.1 (63) was used in R v. 4.1.1 to obtain a t_Nmax_ value for each isolate from the generated growth curves. For sanitizer treatment experiments, isolates were then assigned to one of seven subgroups based on their t_Nmax_ value. Each subgroup was incubated for a different duration, including 36 h, 33.5 h, 31 h, 28.5 h, 26 h, 23.5 h, 21 h, and each given isolate was assigned to the subgroup that represented the closest upper incubation time. For example, an isolate with a t_Nmax_ of 27 h would be assigned to the subgroup incubated for 28.5 h.

### Sanitizer treatment at use-level concentrations

Sanitizer solutions were prepared up to 1 h prior to use. In brief, three sanitizer solutions were prepared in phosphate-buffer saline (PBS), including 80 ppm PAA (Sigma-Aldrich, St. Louis, MO) in PBS adjusted to pH 5.0, 300 ppm BC (Sigma-Aldrich) in PBS adjusted to pH 8.0, and 500 ppm NaOCl (Sigma-Aldrich) in PBS adjusted to pH 6.0. Concentrations of PAA and free chlorine (i.e., hypochlorous acid and hypochlorite ions) in prepared PAA and NaOCl solutions, respectively, were measured using a reflectoquant (RQflex-10; Millipore-Sigma, Burlington, MA) according to the manufacturer’s instructions.

Prior to sanitizer exposure, bacterial cultures were grown to early stationary phase in a 96-well flat-bottom plate. A 200 μL aliquot from each well was subsequently transferred into a 96-deep well plate, which was centrifuged for 10 min at 4,000 rpm. The supernatant was removed, and 12 isolates at a time were resuspended by pipetting up and down eight times with 200 μL of either (i) sanitizer solution for “treated” cells or (ii) PBS for “untreated control” cells; solutions were incubated for 30 s (exposure time includes the time it takes to pipet up and down). For the NaOCl treatment, cell pellets were washed once with PBS prior to exposure with either sanitizer or the control solution. After 30 s incubation, 400 μL of 1.43× Dey-Engley Neutralizing broth (D/E) (BD Diagnostics) was added to both treated and untreated cells, followed by thorough mixing (pipetting up and down eight times) and subsequent incubation for 5 min. Neutralized solutions were serially diluted 1:10 in D/E until a dilution of 10^−9^. All dilutions were spot-plated in 10 μL volume on BHI agar plates, and plates were incubated for 24 h at 37°C, followed by colony enumeration.

### Whole genome sequencing and raw data processing

Bacterial DNA was extracted using DNA extraction kits (Dneasy Blood and Tissue kit and Dneasy Powersoil, Qiagen, Valencia, CA) following the manufacturer’s instructions. Genomic DNA was sequenced using Illumina MiSeq and HiSeq platforms (Illumina, Inc., San Diego, CA) with 2 × 250 bp paired-end reads. The adapters from raw sequencing data were removed using Trimmomatic v 0.36 using default parameters (75), followed by quality assessment using FastQC v 0.11.8 with default parameters (76). Sequences were assembled *de novo* with SPAdes v 3.14.0. with the “—careful” mode; default k-mer sizes were used (77). Quality control of assemblies was performed with QUAST v 5.0.2 (78), and average coverage was determined using SAMtools v 1.11 using an “awk” script (79). Genomes with a minimum of 40× coverage were included in genomic analysis. Contigs smaller than 500 bp were removed, and contigs were classified into taxonomic units using Kraken v 2.1.0 standard database (80) to confirm isolate identity (see Table S1 for WGS data accessibility and NCBI accession numbers for all *Listeria* isolates).

### Genomic analyses

For each species (*L. monocytogenes*, *L. innocua, L. ivanovii, L. seeligeri, L. marthii,* and *L. welshimeri*), kSNP3 v 3.1 was used to identify core SNPs among all assembled genomes, using the optimal k-mer size determined using Kchooser (*k* = 19) (81). For each species, RaxML v 8.2.12 with GTRGAMMAI (82) was used to construct a maximum-likelihood phylogeny using kSNP3 core SNPs as input and 1,000 bootstrap replicates. Trees were visualized and annotated using iTOL (83).

For *L. monocytogenes*, ST and CC were assigned based on identified allelic types (AT) for seven housekeeping genes (i.e., *abcZ, bglA, cat, dapE, dat, ldh,* and *lhkA*) using the Pasteur MLST *L. monocytogenes* database (https://bigsdb.pasteur.fr/cgi-bin/bigsdb/bigsdb.pl?db=pubmlst_listeria_seqdef&page=downloadAlleles). Isolates were grouped into CC unless isolates were assigned a singleton ST; no ST was assigned if a single MLST AT had less than 100% identity and coverage (28).

Genomes were annotated with PROKKA v 1.14.5 (84) using a genetic code of 11 and a minimum contig length of 200 bp. GFF files produced by PROKKA were input into Panaroo v. 1.2.3 (85) to perform pangenome analyses separately for each species, using the parameters “clean-mode strict.” Each core gene alignment produced by Panaroo was queried using SNP-sites v. 2.5.1 (86), generating a core SNP alignment. TreeWAS (24) was used to identify genes and core SNPs associated with log reduction data across each of the three sanitizers, using the Panaroo gene presence/absence matrix and core SNP alignment as input, respectively; the “-c” parameter was applied to only include SNPs that were present in all isolates. TreeWAS was performed on select *Listeria* subpopulations that included more than 20 isolates (i.e., BC and PAA analyses included all *L. monocytogenes* isolates, lineage I, lineage II, lineage III, L. spp., *L. innocua*, *L. seeligeri, L. welshimeri*, and NaOCl analyses included all *L. monocytogenes*, lineage II, and *L.* spp).

Draft assemblies were screened for the presence of several known *Listeria* quat-resistance genes, including *qacE* (87)*, qacH* (*ermC*) (19)*, emrE* (18), *mdrL* (16), the *sugE1/2* operon (*lmo853* and *lmo854*) (50), and *bcrABC* (20), as well as the stress survival islet 2 (SSI-2, *lin0464, lin0465*), which has previously been described to confer tolerance to oxidative stress (33). The sequences for *qacA, qacH, emrE*, *mdrL*, *sugE1, sugE2*, *bcrA, bcrB, bcrC,* and SSI-2 were downloaded from the Pasteur database (https://bigsdb.pasteur.fr/cgi-bin/bigsdb/bigsdb.pl?db=pubmlst_listeria_seqdef&page=downloadAlleles). The gene sequence for *qacE* was obtained from NCBI (NCBI Nucleotide accession no. NZ_AGUG01000015.1). All downloaded sequences were used to create a local nucleotide BLAST database. The database was searched against draft assemblies using BLASTN v. 2.16.0 with default parameters (i.e., a cutoff of >70% sequence identity and maximum e-value of 1e-20). Matches with >90% sequence identity and >90% query coverage indicated the presence of a gene in a given isolate’s draft genome assembly. Genomic data were also analyzed using PLATON v. 5.1 with default parameters to identify the number of plasmids per isolate and the genes that were encoded on plasmids (88).

### Data and statistical analyses

Data were analyzed in R v. 4.1.1. The limit of detection for sanitizer exposure assays was 2 log CFU/mL. In cases where no colonies were observed, the number of bacteria was set to 2 log CFU/mL for statistical analyses. All sanitizer exposure trials were conducted in duplicate. Isolates that were readily available in the lab were included based on convenience in the first experimental replicate. Isolates included for the second experimental replicate were included based on stratified randomization by using R to randomize isolates within their subgroup. In instances where the difference in log reductions obtained for a given isolate was >2 log units between the first and second experimental replicate, a third experimental replicate was performed.

Statistical analysis of data from the sanitizer experiments was performed by fitting linear regression models to log reduction (response variable). For each sanitizer, the explanatory variables included (i) “species”(i.e., *innocua, ivanovii, marthii, monocytogenes, seeligeri,* and *welshimeri*), (ii) “*L. monocytogenes* vs. *L.* spp.,” (iii) “preharvest vs. postharvest,” and (iv) “lineage” (i.e., I, II, and III). *Post hoc* multiple-comparison adjustment was performed with Tukey’s honestly significant differences (HSD) test; alpha level for significance was set at 0.05.

To assess the variance of log reductions achieved with each sanitizer, the Bartlett test of homogeneity of variances was performed on log reduction data for 108 isolates. In addition, a generalized least squares model was fitted to the data with log reduction data as the response variable and sanitizer as the explanatory variable using the nlme package v. 3.1-152 (89). The model also included the constant variance function to obtain estimates of variance. To identify clusters of isolates that showed reduced susceptibility to all three sanitizers, the qtclust function from the flexclust package (90) with a radius of 2 was used. For the qtclust (QTC) analysis, log reduction data for each given sanitizer were averaged for each isolate.

## SUPPLEMENTAL MATERIAL

Table S1 aem.01060-25-s0001.xlsx Metadata and whole genome sequencing (WGS) data accessibility for the 501 *Listeria* isolates included in this study.

Table S2 aem.01060-25-s0002.xlsx SNPs in core genes of *Listeria* subpopulations that were identified as significantly associated with log reductions to benzalkonium chloride (BC), peroxyacetic acid (PAA), and sodium hypochlorite (NaOCl) by three treeWAS association test statistics (i.e., Terminal Score, Simultaneous Score, and Subsequent Score).

ASM does not own the copyrights to Supplemental Material that may be linked to, or accessed through, an article. The authors have granted ASM a non-exclusive, world-wide license to publish the Supplemental Material files. Please contact the corresponding author directly for reuse.
